# The Value and Access of Urban Greenspace: A Comparison Study of User Perceptions of the Naval Cemetery Landscape, New York

**DOI:** 10.3390/ijerph22060870

**Published:** 2025-05-31

**Authors:** Shujuan Li, Alden Stoner, Angela Walseng, Neha Srinivasan, Esther M. Sternberg, Bo Yang

**Affiliations:** 1 School of Landscape Architecture and Planning, The University of Arizona, Tucson, AZ 85721, USA; esternberg@arizona.edu (E.M.S.); boyang17@arizona.edu (B.Y.); 2 Nature Sacred, Annapolis, MD 21401, USA; astoner@naturesacred.org (A.S.); angela@freshtonicmarketing.com (A.W.); nehasrin@umich.edu (N.S.); 3 Andrew Weil Center for Integrative Medicine, College of Medicine, The University of Arizona, Tucson, AZ 85721, USA

**Keywords:** greenspace exposure, self-report health perception survey, urban environment, landscape perception

## Abstract

In studying greenspace and people’s health and wellbeing, the self-report health perception survey method is broadly used. There is a consensus that people’s health and wellbeing are positively associated with greenspace exposure. Meanwhile, different conclusions on details related to greenspace exposure have also been reported, e.g., the frequency and the stay time. Few studies have investigated and compared on-site greenspace exposure and off-site reflections with perspectives on greenspace (i.e., afterward greenspace exposure). Some self-report health perception surveys have been conducted on-site, especially for experimental studies. There are also surveys that have been conducted off-site, e.g., general association studies on greenspace and public health. On-site and off-site settings indicate different time dimensions of greenspace experiences (i.e., real time vs. afterward). To what extent do these survey settings impact the conclusions on greenspace exposure? This study compares visitors’ self-reported health and landscape perceptions of the Naval Cemetery Landscape, a contemplative greenspace for passive recreation activities in Brooklyn, New York. The results show that the on-site survey reached a broader audience, and the perception and valuing of the space captured by the on-site survey were more positive than those of the off-site survey. In addition, the on-site survey captured more details on the associations between greenspace access, perception, and values than the off-site survey did.

## 1. Introduction

There is a wealth of literature supporting the broad spectrum of health and wellbeing benefits associated with greenspace exposure in urban environments [1]. Significant correlations have been identified between urban greenspace visits and use and lowered blood pressure, reduced obesity, reduced risk of cardiovascular disease and other chronic health conditions, and better perceived physical health [2]. Studies have also proved the association between greenspace visits and use and people’s mental health, including a reduction in stress, an improvement in cognitive functioning, and attention restoration [3,4,5]. It is believed that encountering plants and animals in greenspace allows urban residents to recharge or find solitude [6]. Greenspace visits enable rejuvenation, foster contemplation, and offer a sense of peace and calmness [7,8,9]. All these contribute to people’s physical and mental health.

In measuring the health benefits of urban greenspace visits and use, various research designs and measures have been developed. These studies generally fall into two categories based on the research design and the methods of data collection. The first is experimental research. In this type of study, common research setups involve comparing an intervention in a specific greenspace with an intervention in a non-greenspace urban environment [10]. Usually, a small group of experiment participants are recruited and guided to visit/use greenspace and non-greenspace in urban environments. Individual health-related data on neuro-physiological or bodily changes, performance or behavioral changes, and/or self-reported health perceptions are collected to measure the health effects of greenspace visits/use. Most experimental studies measure the perceptions and health effects at the moment of the greenspace visit/use (i.e., on-site).

The second type of research is general association studies. These studies investigate the correlation between public health and greenspace availability. Public health data are usually collected through secondhand information aggregation by local, state, or federal health service departments. Self-report health perception surveys are also broadly used in these association studies [11]. The survey is usually conducted with a broad group of participants at city or regional scales. Most surveys take place off-site from greenspace or without specifying the locations of participation. In many studies, greenspace is defined as a type of land cover rather than a specific location. A correlation analysis is commonly used to examine the relationships between public health indicators, greenspace, and other socio-environmental factors.

A self-report health perception survey assumes that people’s subjective evaluation of their environments serves as a practical and straightforward indicator of the quality of their psychological restoration experiences with greenspace. Scholars argue for the importance of the subjective fit of the environment in supporting the personal goal of developing wellbeing and recovering from mental fatigue [12]. However, a variety of factors may impact people’s subjective perceptions of the health effects of greenspace. Although experimental design research attempts to control other potential variables, general association studies investigate the correlations using a large number of samples.

For both types of research using self-report health perception surveys, temporal factors have seldom been investigated. Some studies have investigated the frequency and duration of greenspace visits and health effects [13]. Studies have reported that visiting greenspace more frequently and spending more time in greenspace were associated with lower perceived stress levels [14,15,16], better mental health and vitality [13,17], and greater wellbeing [18]. In studying the relationship between small urban parks and people’s mental health in Shanghai, China, Wang et al. (2022) found no evidence that the stay time served as a mediator for the relationship between park features and mental health [19]. Hubbard et al. (2021) found that the time duration in greenspace was not associated with general mental health. These conflicting conclusions illustrate the importance of investigating the temporal factors related to people’s perception of greenspace’s health effects [20].

Even less is known about the perception dynamics of the health effects of greenspace over time. Most experimental research collected greenspace experiences on-site and in real time. General association studies rely on recollections of visiting experiences off-site using surveys that unlikely specify the time periods of the greenspace use/visit. In comparison, an on-site survey enables the collection of a sense of landscape that is evoked by a real experience in the field, while an off-site survey usually collects a sense of landscape that is evoked by afterward memories [21]. An on-site survey has the advantage of strong interactivity and can reflect multi-sensory experiences with greenspace. It is usually considered that the most authentic and comprehensive feelings of participants on greenspace can be collected with an on-site survey. In contrast, an off-site survey may involve long delays between the greenspace visit/use and survey completion [21]. Are there differences between the greenspace perceptions of experience at the time of visiting (on-site) and those recollecting greenspace experiences afterward (off-site)? If there are differences, to what extent do the differences impact the conclusions on the health effects of greenspace? These are important questions to better understand the current literature on greenspace and public health.

The goal of this study was to examine the perception differences of a contemplation greenspace between real-time nature exposure and afterward experience recollection. We contrasted the perceptions of people who experienced nature at that moment using an on-site survey with those who had experienced the nature of this site beforehand using an off-site survey. Chi-squared tests were conducted to compare the greenspace perceptions.

## 2. Materials and Methods

### 2.1. Study Site

**Figure 1:**
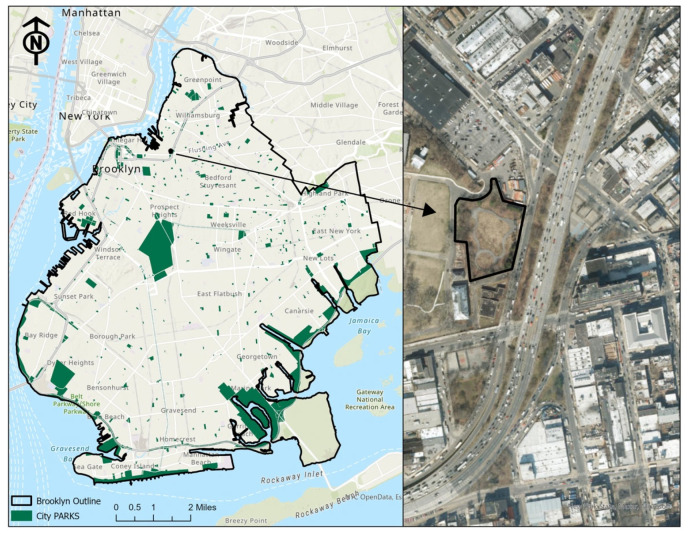
The location of the Naval Cemetery Landscape.

The Naval Cemetery Landscape (NCL) is located in Brooklyn, New York City, and has an area of 1.7 acres (Figure 1). Historically, the site was an unmarked burial ground associated with the Brooklyn Naval Hospital. With funding support from organizations including Nature Sacred (formerly the TFK Foundation), the Brooklyn Greenway Initiative (BGI) designed and restored the abandoned site and turned it into a culturally and ecologically significant public greenspace in 2016. Like other sacred places, this site was designed by the community of primary users of the space, using a charrette process to increase the community’s sense of connection to nature. The design of the NCL was recognized with the Honor Award from the 2020 American Society of Landscape Architects Professional Awards. Today, it is one of the many public spaces that form part of the BGI.

Unlike most urban parks, the NCL was designed not for active recreation but for contemplation. Passing through the gate of the NCL, visitors are immediately transported to a quiet and peaceful place shielded from the surrounding urban environment. Native trees and shrub species line the perimeter of the site, providing a natural screen that blocks views of buildings and the Brooklyn Queens Expressway. Within the park, a sinuous black locust boardwalk connects the Memorial Meadow, Amphitheater, and Sacred Grove. Today, it serves as a space for people to enjoy quiet, quality time and connect with nature. The passive recreation offered by the greenspace makes it a suitable site for studying greenspace perception and nature exposure experiences.

### 2.2. Data Collection

In this study, the survey method was adopted to collect individuals’ subjective landscape and health perceptions of visiting the NCL park. During the survey design, a variety of stakeholders were involved, including Nature Sacred and the BGI. In total, 13 questions were included in the survey, mainly covering the following four topics: demographic information, park access, greenspace use and perceptions, and expectations. In addition to traditional closed-ended and open-ended survey question types, photo and map survey methods were adopted. A pre-test of the survey was conducted on the NCL site with volunteers and staff. The survey123 tool by ESRI was used to build the questionnaire for data collection.

The questionnaire, with three language options (English, Spanish, and Yiddish), was published online. It was distributed with hyperlinks and QR codes and accessible on smart devices with an Internet connection. There was no need to download or install an app on a personal device, thus allowing flexibility and lower costs for public participation. Two sets of hyperlinks and QR codes were created. One set of hyperlinks and QR codes was disseminated on the site of the park and the other set was disseminated by the BGI through its connections off-site. In this way, survey responses from on-site participants and off-site participants could be separated. For the on-site dissemination, multiple versions of flyers were posted on the entrance of the park from 2 June 2023. In addition, iPads with the questionnaire were also accessible for on-site visitors and users. Off-site dissemination was mainly conducted by the BGI. On 11 July, the BGI sent the invitation via email to local residents. Later, the hyperlink to the survey was posted on the webpage of the NCL and included in the monthly/bimonthly newsletters of the BGI.

### 2.3. Data Analysis

IBM SPSS Statistics V29 was used to analyze the survey response data. Two sets of chi-squared tests were conducted. First, the chi-squared goodness of fit test was used to test the statistical significance of differences between on-site and off-site survey responses to the same survey questions. Second, the chi-squared test for independence was conducted to explore the association between participants’ responses to any two survey questions for on-site and off-site survey responses, respectively. An association matrix was developed to present the significance of the associations. For both sets of chi-squared tests, Pearson’s *p* < 0.05 was defined as statistically significant. In addition, a basic descriptive analysis was conducted using Excel. Graphs and tables were produced to summarize the descriptive statistics.

## 3. Results

### 3.1. Total Submissions

**Figure 2:**
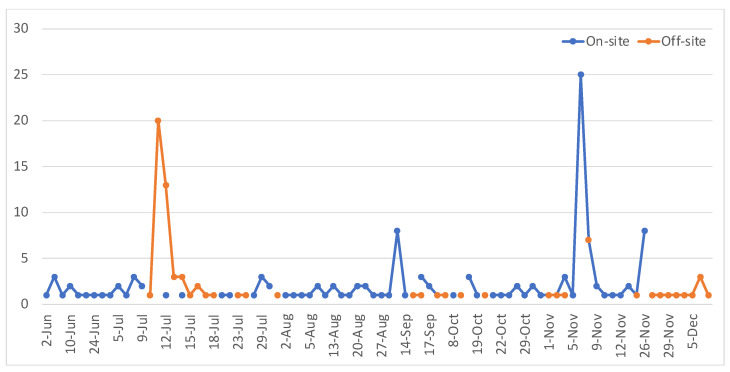
Daily submissions from on-site and off-site participants from June to December 2023.

The dynamic of the survey response counts over the six-month period from June to December 2023 is presented below in Figure 2. In total, 131 on-site survey responses and 74 off-site survey responses were received. With traditional mailed surveys, researchers can count the number of survey questionnaires sent out and the number of responses received. With the survey questionnaire hosted online, and the hyperlinks and QR codes distributed through on-site flyers and off-site emails, it was difficult to calculate the response rates. Considering this is a small park, the total number of survey responses received was solid compared with other similar survey studies of parks [22,23,24,25].

There were different patterns in on-site and off-site survey participation over the six-month period. For on-site participation, the spikes of the number of responses submitted were associated with events in the park and holidays. The off-site survey participation spiked in the first few days after the email dissemination in July. Later, there were few and random survey responses summitted.

### 3.2. Participants’ Profiles

Most of the participants were White or European, representing 64% of the on-site survey participants and 70% of the off-site participants (Table 1). There were few participants from other racial or ethnical groups for both on-site and off-site surveys. For the off-site survey, the percentage of Asian participants was also slightly higher than those using the on-site survey. A much lower percentage of Hispanic and Latino populations participated in the off-site survey compared with the on-site survey. The participants choosing “other” mostly indicated mixed racial or ethnical groups.

Most survey participants were from close-in blocks or Brooklyn (Table 2). There were some differences in terms of living places between on-site participants and off-site participants. The percentage of the on-site participants who were from close-in places (i.e., within a few blocks or Brooklyn) was 71%; it was 86% for off-site participants. Compared with the off-site survey, the on-site survey captured a relatively higher percentage of participants from other NYC boroughs (22% vs. 9%). In addition, the on-site survey successfully recruited participants from other states and overseas. For the visitors with residences outside NYC or from overseas, their last stopping point (e.g., accommodation, hotel, or airport) before travelling to the site might also have been within close-in blocks or Brooklyn, considering that most of them accessed the site by walking or biking.

The chi-squared goodness of fit test results showed that the racial and ethnic groups were not significantly different between the on-site and off-site participants. However, the living places of on-site and off-site participants were significantly different, with a *p*-value below 0.05. We concluded that the two survey methods had reached participants living in different places in this study.

### 3.3. Park Access

Walking and biking were the two major ways that visitors accessed the park. The eight participants who chose “other” ways to arrive at the park all indicated running/jogging in the survey. It was likely that the loop inside the park was on their running/jogging route. Over 80% of the participants accessed the park via physical activities (i.e., walking, jogging, biking, or by scooter or skateboard). The on-site survey showed a lower percentage of participants accessing the park via physical activities than the off-site survey did (77% vs. 86%). This was in line with the fact that the on-site survey also captured participant results from places other than close-in blocks and Brooklyn, including other states and overseas.

For the survey question “On average, how often do you visit this space”, the majority of off-site survey participants considered themselves to be seasonal visitors. In contrast, the majority of the on-site survey participants were seasonal visitors and first-time visitors. On-site survey participants also included countable daily visitors, weekly visitors, and monthly visitors (Table 3).

The place of living impacted the participants’ frequencies of visiting the space. Living closer to the park resulted in people visiting the park more frequently. For both the on-site and off-site survey participants living within a few blocks from the park, 40% indicated that they visited the park daily, weekly, or monthly. The percentage decreased to 17.2% and 13.9% for those living in Brooklyn or other NYC boroughs, respectively. The majority of participants who lived in NYC (within a few blocks, Brooklyn, or other NYC boroughs) considered themselves to be seasonal visitors to the park. For participants outside NYC, most were first-time visitors to the park.

On-site participants living close to the park showed more frequent visits than off-site participants did. For those living within a few blocks of the park, 46% visited the park frequently (daily, weekly, or monthly) based on the on-site participants’ responses. This percentage decreased to 22% for off-site participants. In total, 19.4% of Brooklyn on-site participants indicated that they visited the park frequently. The percentage was 14.5% for Brooklyn off-site participants. There were 17% participants from other NYC boroughs who considered themselves to be frequent park visitors based on the on-site survey. In contrast, 0 out of 7 off-site participants from other NYC boroughs visited the park frequently.

### 3.4. The Perception and the Value of the Greenspace

Both on-site and off-site participants valued greenspace. There were no significant differences between on-site and off-site participants in their responses to survey questions about perceptions of greenspace based on the chi-squared goodness of fit tests. Survey participants were asked to select two “must haves” within close walking distance of home; greenspace and grocery stores were the two most selected, and greenspace was ranked slightly higher than grocery stores by both on-site participants and off-site participants. In total, 87% of on-site participants and 84% of off-site participants selected greenspace as one of the two “must haves”. Other choices (i.e., pharmacy, coffee shop, and dining options) received a much lower percentage of selection from the survey participants.

**Figure 3:**
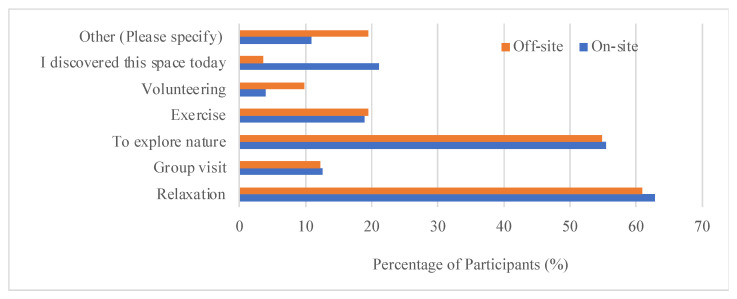
The purposes of typical visiting by on-site and off-site survey participants.

For this specific greenspace, survey participants were asked “How do you view the importance of spaces like this?”. Almost all participants valued it as “an essential to individual and public health” or “a nice to have amenity”. Only 3 survey participants selected “doesn’t matter” or “waste of resources”. The on-site participants valued this greenspace more than the off-site participants. In total, 81% of on-site participants and 73% of off-site participants identified this greenspace as being essential to individual and public health. Specifically, participants most valued this space for relaxation and exploring nature. Both on-site participants and off-site participants presented similar patterns for the purposes of visiting this greenspace. Other than “relaxation” and “to explore nature”, other choices (including “exercise”, “volunteering”, and “group visit”) were much less selected by participants (Figure 3).

**Figure 4:**
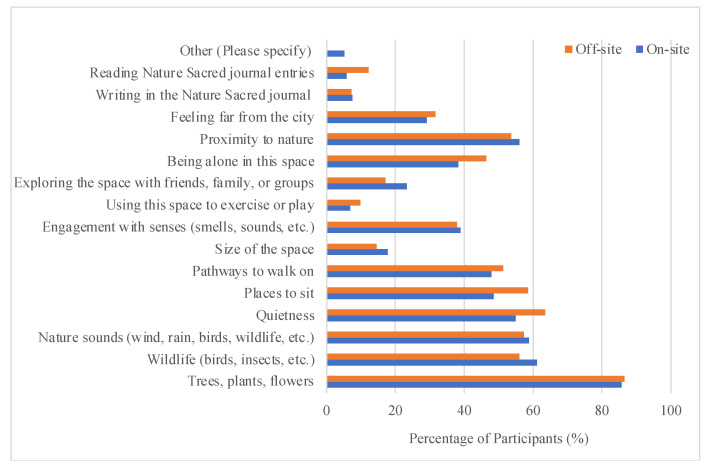
The features giving the greatest sense of relaxation and/or rejuvenation in the park based on on-site and off-site survey participant responses.

A variety of landscape features and the design of this space were valued by the survey participants. For this multiple-choice question, the answer choices included major landscape features in the park, highlighted design objects, and frequently observed space-use activities by the stakeholders and the park management volunteers. The survey results showed that “Trees, plants, flowers” were the most favored by survey participants (Figure 4). In total, 85% of the participants chose them as the landscape features that gave them the greatest sense of relaxation and/or rejuvenation in the space. “Quietness”, “wildlife”, “nature sound”, “proximity to nature”, “pathways to walk on”, and “places to sit” were also highly valued. The off-site participants and the on-site participants had similar preferences for the existing landscape features and design of the space. “Using this space to exercise or play” did not receive much attention. This was in line with the design of this space for contemplation, not for active recreation.

**Figure 5:**
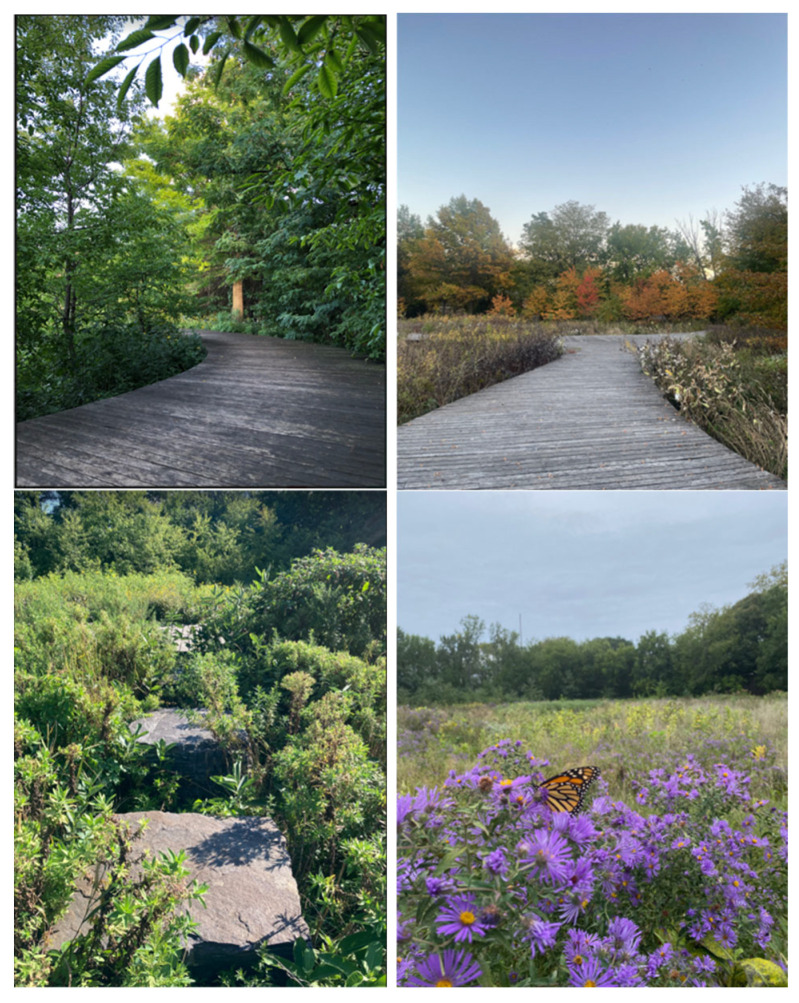
Selected pictures of a view or spot that survey participants enjoyed most.

Participants were also asked to submit a picture of a view or spot within the space they enjoyed most. Thirty pictures were submitted with the on-site surveys and four pictures with the off-site surveys. Using a visual inspection, we ascertained that the picture contents were in line with the results of the above survey question on favorite landscape and design features. The pictures were filled with trees, plants, flowers, and pathways. The quietness and proximity to nature were imageable through those pictures (Figure 5).

**Figure 6:**
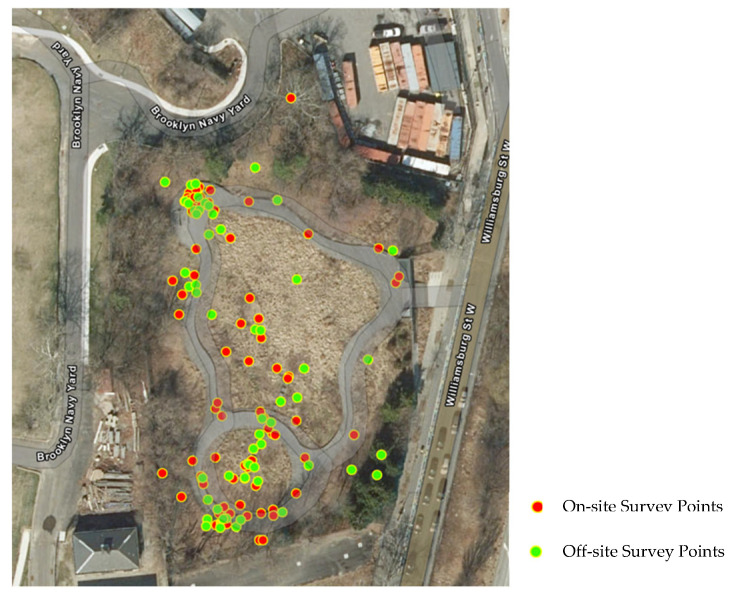
The favorite spots of survey participants in the park.

Spatially, participants liked the locations away from the entrance and locations with favorite designed landscape features. The survey asked participants to place a pin on the map over their favorite spot. The results are shown on the map below (Figure 6). The hot spots of the on-site and off-site participants’ responses were distributed around the amphitheater, the stone blocks, the sacred grove, and the bench.

**Figure 7:**
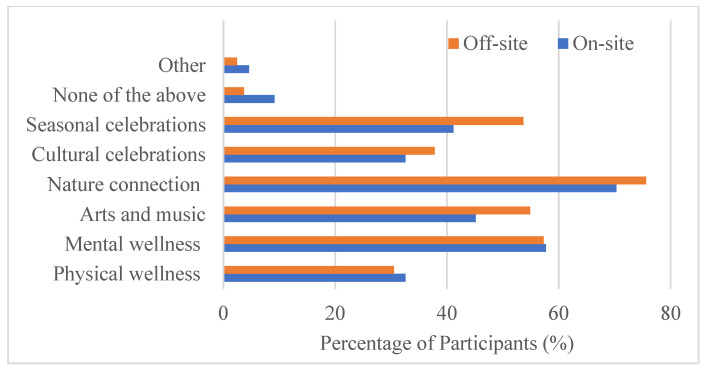
The wellness programs that survey participants were interested in.

For this space, survey participants expressed interest in a variety of wellness programs (Figure 7). For both on-site and off-site participants, nature connection (bird watching, nature walks, community planting events, etc.) was the top choice, with around 70% of survey participants’ support, followed by mental wellness (horticultural therapy, counseling, meditation sessions, sound bathing, etc.), seasonal celebrations, and arts and music (painting, sketching, concerts, dance, theater, etc.). Physical wellness (yoga, Zumba, etc.) and cultural celebrations also gained support from around 30% of the survey participants.

**Figure 8:**
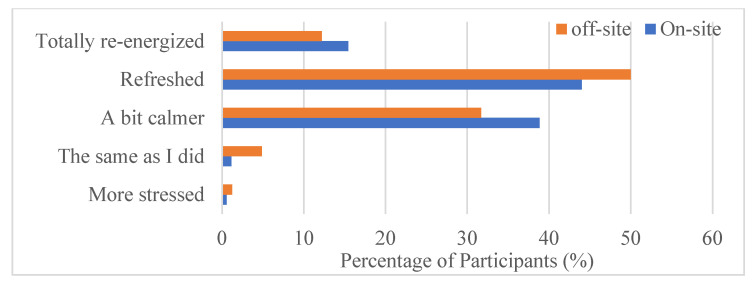
The impact on individual stress levels by visiting this space.

In terms of mental health effects, the survey results showed that visiting this greenspace reduced participants’ stress levels, with 50% of participants reporting feeling refreshed and almost 35% of participants feeling a bit calmer (Figure 8). Over 10% of participants felt totally re-energized from visiting the park for both the on-site survey and the off-site survey. A higher percentage of on-site participants than off-site participants felt totally re-energized. Five off-site and two on-site participants indicated that they felt the same as they did before their visit to the park.

### 3.5. The Associations between Access, Perception, and the Value of this Greenspace

To assess the associations between access, perception, and the value of this space by visitor, the chi-squared test for independence was adopted; *p* < 0.05 was defined as statistically significant. The results showed various associations among access, perception, and the value of greenspace in this study. A matrix of the associations’ *p*-values was developed for on-site and off-site participants’ responses, respectively (Table 4 and Table 5). For the on-site survey responses, 16 significant associations were detected among access, perception, and the value of greenspace, highlighted in bold text in the table. In contrast, only three significant associations could be identified from off-site survey responses.

Based on the on-site survey results, the place of living was significantly associated with the frequency of visiting and the way of accessing the park. The way of accessing the park was significantly associated with participants’ views on greenspace in general (i.e., the survey question on “must haves”) and this park specifically (i.e., the survey question “How do you view the importance of spaces like this?”). The way of accessing the park was also significantly associated with the purposes of visiting this park and the health effects they perceived with visiting the park. In addition, a significant association was detected between the way of accessing the park and the wellness programs that the on-site participants were interested in. The views on greenspace in general and the views on this specific park were significantly positively associated, and both were also significantly positively associated with the health effects that on-site participants reported on visiting the park. In summary, the ways of access, reported health effects, views on greenspace in general, and views on this park specifically were highly related to each other and other factors.

In contrast, there were only three significant associations detected from the off-site survey responses. These were the associations between the way of accessing the park and the purpose of the park visit, between the views on this park specifically and the perceived health effects, and between the purposes of the park visit and the views on greenspace in general. These three significant associations covered park access, perception of greenspace, and the value of park visiting.

## 4. Discussion

### 4.1. Survey Methods

With the development of cloud services, survey questionnaires can be easily developed online and reach a large group of participants. However, few studies have investigated the potential impacts of this method on survey results and research conclusions. In this study, on-site and off-site survey methods reached different audiences. The on-site survey captured the voices of participants from a variety of places, including visitors from other states and overseas. Many of the visitors were visiting the study site for the first time. For the off-site survey, most participants were from Brooklyn. As a result, their access to the park and visiting behavior were quite different. This result was similar to other related studies. For example, Guimarães et al. (2015) studied birdwatchers’ preferences using survey methods. They found that their on-site survey was able to contact new visitors, while their off-site survey reached former visitors [26].

In addition, studying landscape perceptions can also be impacted by the survey methods adopted. In this study, the on-site survey collected participants’ opinions based on their real-time experience and the off-site survey collected participants’ opinions based on their previous experiences of visiting the park. The real-time experience and experience memories of greenspace use/visit can reflect different aspects of landscape preferences and values. In this study, the chi-squared analysis results showed that the overall perception and value of greenspace were similar but the on-site survey captured more details on the associations between greenspace access, perception, and values than the off-site survey did.

Although survey methods have been broadly adopted in greenspace studies, this study calls for the investigation of potential impacts on study results caused by different survey methods. As indicated in this study, different survey methods may result in the inclusion of different audiences, reflecting different aspects of experiences and preferences related to greenspace. Other potential impacts on the study results caused by the survey methods employed need further investigation.

### 4.2. Real-Time Experience vs. Previous Experience Reflection

A large number of studies have been conducted on the health benefits of greenspace experiences. Controlled experiments are broadly adopted to measure health benefits during or immediately after visiting greenspace. The survey method is also extensively used to collect self-reported health benefits or landscape perceptions during greenspace use. Few studies have compared benefits and perceptions based on real-time experiences and previous experience reflections. In this study, real-time greenspace experiences were collected using an on-site survey, and previous greenspace experiences were reflected on using an off-site survey. Chi-squared good of fitness tests showed that there were no significant differences on park perception and value between real-time greenspace experiences and previous greenspace experience reflections. Relatively, the perception of greenspace was more positive for real-time nature exposure based on the on-site survey than for the previous greenspace experience reflections collected using the off-site survey.

In addition, the survey results of the real-time greenspace experience revealed more associations among access, greenspace perception, and greenspace value than previous greenspace experience reflections did. Although many studies have been devoted to understanding the factors that may impact the health benefits of nature exposure, it is important to differentiate the experiences of real-time usage and general perceptions afterward of greenspace visits. Based on this study, it seemed that afterward reflections of greenspace experiences only retained a limited number of key factors. From a planning and design perspective, it is important to highlight key factors in order to promote greenspace visits/use. Although some mental health benefits may not be measurable or memorable, benefiting from the services provided by greenspace is dependent upon usage. Understanding the differences can inform efforts in the design and management of greenspace in ways that enhance the use of greenspace and maximize positive impacts on wellbeing and quality of life.

### 4.3. Implications for the Design of Nature Spaces for Mental Health and Wellness Benefits

In dense urban areas, greenspace is a limited resource and contemplative greenspace is even scarcer. In places like Brooklyn, even when stepping outside into areas with greenery, it remains challenging to escape the pervasive built environment. A lack of exposure to biodiverse nature environments has been linked to mental health problems. Different from major urban parks designed for active recreation, contemplative greenspace is designed for passive exposure and connection to nature, ultimately leading to the psychological or mental states of contemplation. Scholars have investigated landscape designs that create a contemplative outdoor space [27,28]. So far, there have been few studies investigating the performance of contemplative greenspace.

The NCL was designed and built as a contemplative greenspace, a site that supports activities requiring a minimal use of facilities and with a low environmental impact. Contemplation can be achieved by simply relaxing and being in nature, which seems to be the main motivation for visiting contemplative parks by urban dwellers [27]. As identified in this study, the major purposes of visiting this greenspace by survey participants were relaxation and to explore nature. The landscape features and design of this space that were most valued by survey participants were the nature characteristics (vegetation, wildlife, nature sound and quietness, etc.). The mental health effects of visiting this place were positive, based on the survey results. Almost all survey participants indicated that they felt totally re-energized, refreshed, or a bit calmer. Only 5% of participants indicated that they felt the same as or more stressed than they did before their visit to the park. For future wellness programs for this greenspace, survey participants ranked nature connections as the program they would most like to join.

The success of the NCL provides an excellent example for developing contemplative greenspace in urban areas. Small, community-led, and biodiverse greenspace can provide an accessible and effective place for contemplation in dense urban environments. Previous studies have shown that small parks are a successful type of urban park, attracting visits and serving local residents [29]. Easy access and proximity can encourage visits, especially from vulnerable populations [30]. Scholars have called for the development of more small parks [31]. Investment in additional small contemplative parks could benefit local residents’ mental health and wellbeing and provide a viable solution to the shortage and the lack of accessible urban parks at the same time.

### 4.4. Under-Represented Populations

Although our findings showed that this space was successful, we also noted low participation in the surveys from minorities. The majority of participants for both the on-site and the off-site surveys were white (64% and 70%, respectively). In contrast, white residents accounted for only 23% to 53% of the total population in nearby neighborhoods (Table 6). Although 6% to 44% of the population in the surrounding neighborhoods are Black and 8% to 26% are Hispanic and Latino, only 10 participants identified themselves as Black (6 on-site and 4 off-site) and 10 participants identified themselves as Hispanic or Latino (9 on-site and 1 off-site).

The surrounding communities are diverse in race, ethnic identities, religion, and culture. The Williamsburg–South Side neighborhood is close to the study area and is mainly occupied by the Yiddish-speaking Hasidim (predominantly Satmar Hasidim). Spanish is also broadly identified as a language spoken at home in the surrounding neighborhoods. As the languages used in a survey questionnaire may impact survey participant recruitment and the inclusiveness of the research, this study offered the survey questionnaires with English, Spanish, and Yiddish language options. Survey flyers were also shared in multiple languages. However, no responses were submitted in Spanish nor Yiddish. This was in line with other similar survey studies. More research is needed on exploring the causes for low responses in languages other than English in multilanguage communities.

The low representation of non-white and non-English-speaking survey participants may have been caused by multiple factors. One reason could have been low NCL greenspace visits and use by the minority population groups. As indicated by other studies, people with a lower socioeconomic status and people from ethnic minority backgrounds are under-represented in greenspace visits/use due to personal, social, physical, and other factors [32,33]. Another reason could have been low interest in survey participation by the minority population groups. This is a phenomenon broadly identified in other research areas, e.g., health studies [34,35]. In this study, the off-site survey was disseminated to local residents by the BGI. We assumed that local residents had equal opportunities to participate in the survey. The low interest in the NCL and participating in the survey may have contributed to the low response rate from minority groups.

## 5. Conclusions

In summary, this study compared visitors’ self-reported health and landscape perceptions of the NCL, a contemplative greenspace in Brooklyn, New York. Both on-site and off-site survey results indicated that the design of the NCL was highly successful at achieving the mental health goals of this greenspace for contemplation, stress reduction, and restoration. The study identified specific design features and locations of these features that were particularly restorative for visitors (e.g., away from the entrance, rich in biodiversity and nature, and quiet spaces). At the same time, the results showed that the on-site survey reached a broader audience, and the perception and valuing of the space captured by the on-site survey were more positive than those of the off-site survey. In addition, the on-site survey captured more details on the associations between greenspace access, perception, and values than the off-site survey. Finally, this study provides a model for survey methodology for future studies of such spaces.

## Figures and Tables

**Table 1:** The racial and ethnic groups of survey participants.

	On-Site	Off-Site
Asian or Asian American	8	6
Black or African American	6	4
Hispanic or Latino	9	1
Middle Eastern or North African	4	2
Other	8	5
Prefer Not To Say	12	4
White or European	84	52
Grand Total	131	74

**Table 2:** Typical ways for on-site and off-site survey participants to access the park.

**On-site**	Walk	Bike	Scooter or Skateboard	Bus	Subway	Taxi or Rideshare	Personal Vehicle	Other	**Total**
Within a few blocks	19	5	1					1	26
Brooklyn	22	27		5	1	2	8	2	67
Another NYC borough (Bronx, Manhattan, Queens, Staten Island)	6	11		1	6		4	1	29
Other areas outside NYC	4	2					1		7
Outside of the United States	1						1		2
**Total**	52	45	1	6	7	2	14	4	131
**Off-site**	Walk	Bike	Scooter or Skateboard	Bus	Subway	Taxi or Rideshare	Personal Vehicle	Other	**Total**
Within a few blocks	5	3	1						9
Brooklyn	18	27		3	1		2	4	55
Another NYC borough (Bronx, Manhattan, Queens, Staten Island)		4			1		2		7
Other areas outside NYC in the		2					1		3
**Total**	23	36	1	3	2		5	4	74

**Table 3:** The living locations of on-site and off-site survey participants and frequencies of visiting.

**On-site**	Daily	Weekly	Monthly	Seasonally	First time visitor	**Total**
Within a few blocks	2	2	8	9	5	26
Brooklyn		5	8	37	17	67
Another NYC borough (Bronx, Manhattan, Queens, Staten Island)		3	2	12	12	29
Other areas outside NYC				1	6	7
Outside of the United States					2	2
Total	2	10	18	59	42	131
**Off-site**	Daily	Weekly	Monthly	Seasonally	First time visitor	**Total**
Within a few blocks			2	6	1	9
Brooklyn		2	6	39	8	55
Another NYC borough (Bronx, Manhattan, Queens, Staten Island)				5	2	7
Other areas outside NYC				2	1	3
**Total**		2	8	52	12	74

**Table 4:** The *p*-value matrix of access, perception, and value associations based on the on-site survey.

**On-site**	Place Live	Visit Frequency	Ways of Access	Time Visit	Visit Purpose	Health Effects	Wellness Program	View Specific	View General	Design Features
Place Live		**0.006**	**0.016**	0.17	0.574	0.478	0.998	0.376	0.568	0.665
Visit Frequency			0.67	**0.029**	**0.009**	0.384	0.99	0.536	0.977	0.295
Ways of Access				0.837	**0.034**	**<0.001**	**<0.001**	**<0.001**	**<0.001**	0.221
Time Visit					0.06	0.391	0.218	0.282	0.185	0.563
Visit Purpose						0.99	0.681	0.999	0.061	0.587
Health Effects							**<0.001**	**<0.001**	**<0.001**	0.144
Wellness Program								**<0.001**	0.649	**0.004**
View Specific									**<0.001**	0.945
View General										**0.02**
Design Features										

Note: Bold values are *p* < 0.05.

**Table 5:** The *p*-value matrix of access, perception, and value associations based on the off-site survey.

**Off-site**	Place Live	Visit Frequency	Ways of Access	Time Visit	Visit Purpose	Health Effects	Wellness Program	View Specific	View General	Design Features
Place Live		0.889	0.052	0.424	0.142	0.943	0.702	0.884	0.98	0.186
Visit Frequency			0.572	0.615	0.587	0.585	0.87	0.428	0.996	0.277
Ways of Access				0.694	**<0.001**	0.942	0.635	0.895	0.987	0.52
Time Visit					0.965	0.537	0.165	0.773	0.73	0.52
Visit Purpose						0.63	0.118	0.187	**0.012**	0.428
Health Effects							0.66	**<0.001**	0.123	0.245
Wellness Program								0.769	0.563	0.227
View Specific									0.422	0.316
View General										0.89
Design Features										

Note: Bold values are *p* < 0.05.

**Table 6:** The population and races of nearby neighborhoods.

Neighborhoods	Total Population	White	Hispanic	Black	Asian	Others
Navy Hill	32,714	52.5%	7.5%	14.5%	10%	15.50%
Clinton Hill	29,868	40.7%	10.7%	22.6%	7.6%	18.40%
Bedford-Stuvysant	200,310	23.4%	16.7%	44.1%	4.8%	11.00%
Williamsburg-South Side	161,169	52.3%	26.1%	6.4%	7.9%	7.30%

## Data Availability

Survey data is unavailable due to privacy or ethical restrictions.
